# Myocardial injury and COVID-19: Serum hs-cTnI level in risk stratification and the prediction of 30-day fatality in COVID-19 patients with no prior cardiovascular disease

**DOI:** 10.7150/thno.47980

**Published:** 2020-07-29

**Authors:** Jiatian Cao, Yan Zheng, Zhe Luo, Zhendong Mei, Yumeng Yao, Zilong Liu, Chao Liang, Hongbo Yang, Yanan Song, Kaihuan Yu, Yan Gao, Chouwen Zhu, Zheyong Huang, Juying Qian, Junbo Ge

**Affiliations:** 1 Department of Cardiology, Zhongshan Hospital, Fudan University. Shanghai Institute of Cardiovascular Diseases. 180 Feng Lin Road, Shanghai 200032, China.; 2 State Key Laboratory of Genetic Engineering, School of Life Sciences, Fudan University, Shanghai 200438, China.; 3 Ministry of Education Key Laboratory of Public Health Safety, School of Public Health, Fudan University, Shanghai, 200438 China.; 4 Department of Critical Medicine, Zhongshan Hospital, Fudan University, 180 Feng Lin Road, Shanghai 200032, China.; 5 Department of Infectious Diseases, Zhongshan Hospital, Fudan University, 180 Feng Lin Road, Shanghai 200032, China.; 6 Department of Pulmonary and Critical Care Medicine, Zhongshan Hospital, Fudan University, 180 Feng Lin Road, Shanghai 200032, China.; 7 Department of Anesthesiology, Zhongshan Hospital, Fudan University, 180 Feng Lin Road, Shanghai 200032, China.; 8 Department of Hepatobiliary Surgery, Renmin Hospital of Wuhan University. Gaoxin 6th Road, Donghu high tech Development Zone, Wuhan 430200, China.; 9 Department of Neurology, Remin Hospital of Wuhan University, 99 Ziyang Road,Wuchang District, Wuhan 430200, China.; 10 Department of Gastroenterology, Zhongshan Hospital, Fudan University, 180 Feng Lin Road, Shanghai 200032, China.

**Keywords:** Troponin I, COVID-19, myocardial injury, in-hospital fatality

## Abstract

**Introduction:** To explore the involvement of the cardiovascular system in coronavirus disease 2019 (COVID-19), we investigated whether myocardial injury occurred in COVID-19 patients and assessed the performance of serum high-sensitivity cardiac Troponin I (hs-cTnI) levels in predicting disease severity and 30-day in-hospital fatality.

**Methods:** We included 244 COVID-19 patients, who were admitted to Renmin Hospital of Wuhan University with no preexisting cardiovascular disease or renal dysfunction. We analyzed the data including patients' clinical characteristics, cardiac biomarkers, severity of medical conditions, and 30-day in-hospital fatality. We performed multivariable Cox regressions and the receiver operating characteristic analysis to assess the association of cardiac biomarkers on admission with disease severity and prognosis.

**Results:** In this retrospective observational study, 11% of COVID-19 patients had increased hs-cTnI levels (>40 ng/L) on admission. Of note, serum hs-cTnI levels were positively associated with the severity of medical conditions (median [interquartile range (IQR)]: 6.00 [6.00-6.00] ng/L in 91 patients with moderate conditions, 6.00 [6.00-18.00] ng/L in 107 patients with severe conditions, and 11.00 [6.00-56.75] ng/L in 46 patients with critical conditions, *P* for trend=0.001). Moreover, compared with those with normal cTnI levels, patients with increased hs-cTnI levels had higher in-hospital fatality (adjusted hazard ratio [95% CI]: 4.79 [1.46-15.69]). The receiver-operating characteristic curve analysis suggested that the inclusion of hs-cTnI levels into a panel of empirical prognostic factors substantially improved the prediction performance for severe or critical conditions (area under the curve (AUC): 0.71 (95% CI: 0.65-0.78) vs. 0.65 (0.58-0.72), *P*=0.01), as well as for 30-day fatality (AUC: 0.91 (0.85-0.96) vs. 0.77 (0.62-0.91), *P*=0.04). A cutoff value of 20 ng/L of hs-cTnI level led to the best prediction to 30-day fatality.

**Conclusions:** In COVID-19 patients with no preexisting cardiovascular disease, 11% had increased hs-cTnI levels. Besides empirical prognostic factors, serum hs-cTnI levels upon admission provided independent prediction to both the severity of the medical condition and 30-day in-hospital fatality. These findings may shed important light on the clinical management of COVID-19.

## Introduction

Coronavirus disease 2019 (COVID-19), an infectious disease caused by a novel SARS-coronavirus 2 (SARS-CoV-2), is an ongoing global health threat. Based on World Health Organization's data on July 17^th^, 2020, there were more than 13.7 million confirmed cases and about 0.58 million deaths in 216 countries and regions 1. According to the latest data from the Chinese Center for Disease Control and Prevention, 72,314 confirmed cases were reported in China 2. Among them, 10.5% of COVID-19 patients had comorbid cardiovascular diseases 2. Preexisting cardiovascular disease is one of risk factors for poor prognosis in COVID-19 patients 2-5. Meanwhile, the circulating levels of cardiac troponin I (cTnI), a cardiac biomarker specific to myocardial injury, were positively associated with the preexisting medical conditions, especially cardiovascular disease and renal dysfunction 6. Moreover, a few studies have reported that myocardial injury was a common clinical feature in critically ill COVID-19 patients 4, 7, 8. Interestingly, angiotensin-converting enzyme 2 (ACE2), the functional receptor of SARS-CoV-2, was highly expressed in vascular endothelial cells and myocardium 9-11, implying the potential link between the cardiovascular system and COVID19.

In clinical practice, however, it is challenging to determine whether the elevated cTnI levels are caused by direct COVID-19 infection or by the progress of these preexisting medical conditions 12. Thus, a lot of key questions remain elusive. For example, we do not know whether SARS-CoV-2 would attack the heart via ACE2 or not, currently, no data is available to address whether myocardial damage has any effect on clinical prognosis (if so, to what extent), especially after the exclusion of COVID-19 patients with preexisting cardiovascular disease 9, 13.

To address these key questions, we hypothesized that higher hs-cTnI levels caused by SARS-CoV-2 infection is associated with a more severe medical condition and poorer prognosis in COVID-19 patients. To test this hypothesis, we collected data from 244 COVID-19 patients without preexisting cardiovascular disease in Renmin Hospital of Wuhan University, investigated the clinical features focusing on cardiac impairments caused by COVID-19, and assessed the prediction performance of serum hs-cTnI levels for disease severity and 30-day in-hospital fatality.

## Methods

### Study design and patients

**Figure 1:**
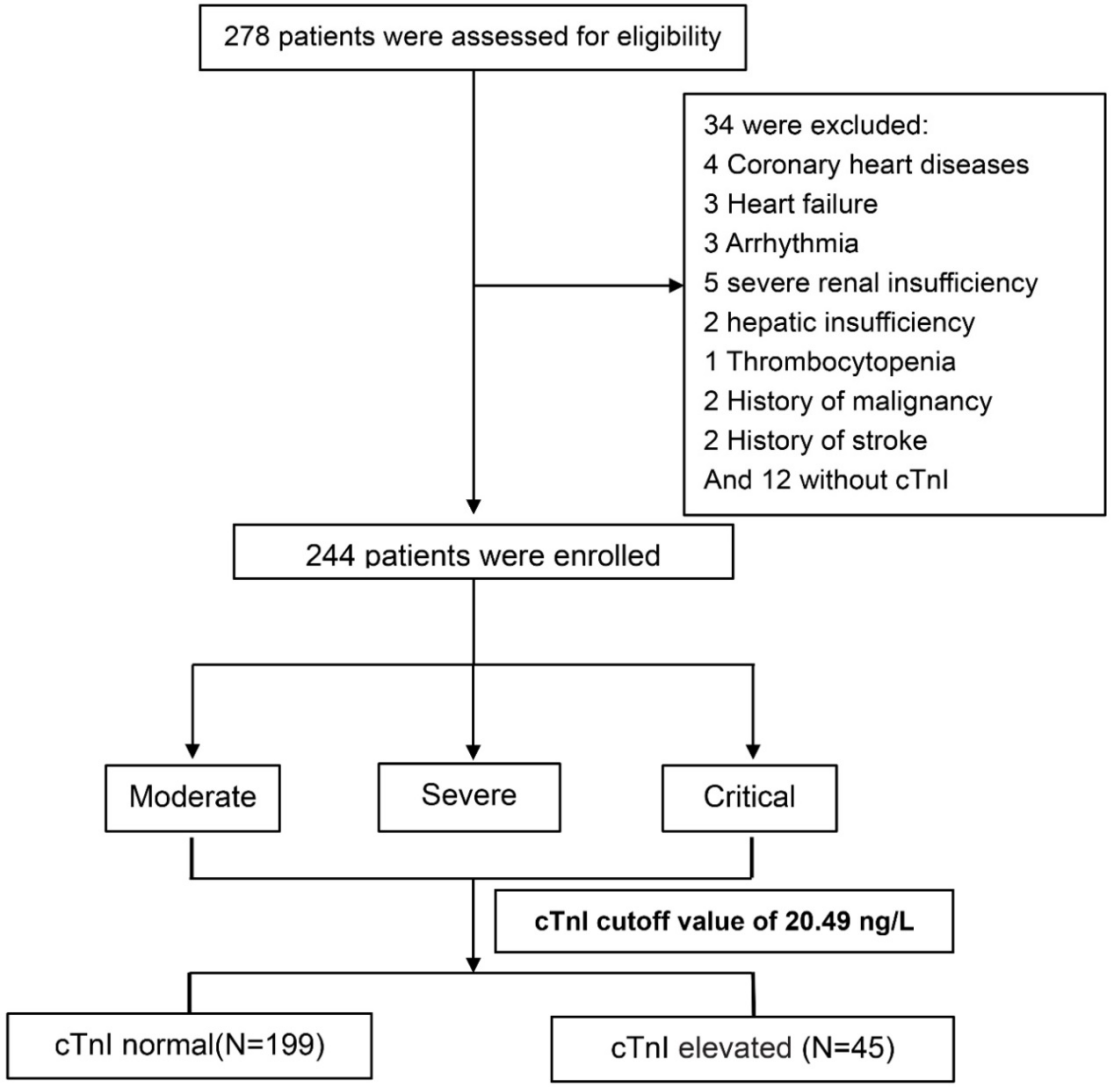
Flowchart of the participants' enrollment, classification, and follow-up. Abbreviations: hs-cTnI, high-sensitivity cardiac troponin I.

We assessed 278 consecutive COVID-19 patients who were admitted to the negative-pressure special wards which were reformed to provide respiratory support and intensive care, Renmin Hospital of Wuhan University from February 6 to February 21, 2020. The current cohort excluded patients with coronary heart diseases (n=4), heart failure (n=3), severe arrhythmia (n=3, e.g., atrial fibrillation, frequently premature ventricular contractions, ventricular tachycardia), severe renal insufficiency (n=5, defined as eGFR<30 ml/min/1.73 m^2^), severe hepatic insufficiency (n=2, defined as alanine transaminase or aspartate transaminase >3 times upper limit of normal range), thrombocytopenia (n=1), history of malignancy (n=2), and/or stroke (n=2), and patients without hs-cTnI measurements (n=12) (**Figure 1**). In total, we enrolled 244 COVID-19 patients into this study and collected their data retrospectively. This study was approved by the institutional Ethics Committee of Renmin Hospital of Wuhan University (Wuhan, China).

In the current study, all COVID-19 patients were diagnosed according to World Health Organization interim guidance 14. The medical condition of patients (i.e., mild, moderate, severe, and critical) were defined by the Novel Coronavirus-Infected Pneumonia Diagnosis and Treatment (Provisional 6th Edition) released by the National Health Commission of China (Supplemental Method). Briefly, patients were defined as mild cases if they had mild symptoms without pneumonia manifestation in imaging test; as moderate cases if they had respiratory tract symptoms and pneumonia manifestation by imaging; as severe cases if they had any of the following situations: respiratory rate >30/min, oxygen saturation <93%, PaO2/FiO2 ratio <300 mmHg, patients with >50% lesions progression within 24 to 48 hours by pulmonary imaging; or as critically ill cases if they had respiratory failure requiring mechanical ventilation, shock, or respiratory failure combined with other organ failure requiring ICU treatment.

After the preliminary screening, most of the patients transferred to Renmin Hospital of Wuhan University were in moderate, severe, or critical conditions. Medical history information was obtained from patients and their families during hospitalization using a standardized tabular questionnaire. Laboratory tests and chest CT or X-ray were conducted on admission, including complete blood cell and neutrophil counts, hs-cTnI, C-reactive protein (CRP), creatinine kinase, creatinine kinase-myocardial band (CK-MB), N-terminal pro-B-type natriuretic peptide (NT-proBNP), serum biochemistry, and respiratory pathogens testing, such as influenza A virus, influenza B virus, and adenovirus 6, 8. Serum hs-cTnI levels were assessed by a two-site sandwich chemiluminescence immunoassay via DVIA Centaur TnI-Ultra laboratory system (ADVIA Centaur XP, Siemens Healthcare Diagnostics, Erlangen, Germany). The detection range of this assay was 6-50000 ng/L, wherein the 99^th^ percentile of a healthy population is 40 ng/L, which was the standard cut-off value for myocardial injury in clinical practice. Unmeasurable levels of hs-cTnI were imputed as 6 ng/L (the lower limit of detection). The measurement of NT-proBNP was based on a double-antibody clip one-step enzyme immunoassay and the detection instrument was a Dimension EXL with LM automatic biochemical analysis system.

### Statistical analysis

Patients were categorized into three groups according to the severity of medical conditions (Supplemental Method). Categorical variables were expressed as frequencies (percentage), and continuous variables as mean ± standard deviation (SD) or median (interquartile range [IQR]). Clinical characteristics and laboratory markers were compared across different groups of patients. *P* for trend across three groups was calculated using linear or logistic regressions. Continuous variables were log-transformed before analysis when they showed right-skewed distributions. We conducted a survival analysis with the dependent variable of time to fatality, setting time zero to day of hospital admission. Patients were considered right-censored if they were 1) discharged from the hospital alive or 2) remained in the hospital at the time of data freeze (March 22, 2020). The Kaplan-Meier method was used to estimate the cumulative death rate and the stratified log-rank statistic to compare the time-to-event endpoints stratified by hs-cTnI group. The Cox proportional hazards regression models were used to estimate hazard ratio for death associated with the hs-cTnI grade, after controlling for the aforementioned empirical prognostic factors. To assess the prediction performance of hs-cTnI levels on admission for the severity of medical conditions, and for in-hospital fatality risk, the area under the receiver operator characteristic (ROC) curves (AUC) was calculated in hierarchical models. In consideration of the previous clinical evidence and the feasibility of clinical practice, the basic model included empirical prognostic factors 15-19 of age, sex, hypertension, diabetes, and renal function measured by eGFR. Other models were built on top of the basic model, by further including hs-cTnI levels or clinical severity condition. The optimal statistical cut-off value for hs-cTnI levels was calculated based on ROC curve as the maximum (sensitivity + specificity - 1) in the univariable analysis 20. Sensitivity, specificity, and positive and negative likelihood ratios were assessed for optimal cutoffs obtained from our ROC curve analysis. Because the biomarker of hemodynamic stress (i.e., NT-proBNP) was measured in the patients with a high risk of cardiac dysfunction, we further assessed the prediction performance of NT-proBNP for prognosis as a secondary analysis in the patients with data available. Two-sided *P* values <0.05 were considered statistically significant. All statistical analyses were performed using R version 3.5.1 (https://www.r-project.org/).

## Results

### Patient characteristics on admission

Among 244 patients in our analysis, 133 (54.5%) were males, and the mean (SD) age on admission was 62.58 (13.43) years, and the median time since symptom onset was 10 days (IQR: 8-14). The level of hs-cTnI was detectable in 88 (36.1%) COVID-19 patients and the median (IQR) hs-cTnI level was 6 (6-12.8) ng/L. In our study, 100 patients (41.0%) had preexisting medical conditions (75 hypertensive patients, 36 diabetes patients, 11 patients with both hypertension and diabetes). For the COVID-19 severity assessment, 91 patients (37.3%) were with moderate conditions, 107 (43.9%) with severe conditions, and 46 (18.9%) with critically ill conditions (**Table 1**).

### Comparison of clinical features across disease severity groups

More severe medical condition was associated with older age (the mean [SE] age was 59.79 [13.49] years, 62.20 [13.43] years, and 69.89 [11.26] years for those in moderate, severe, and critical conditions, respectively), a higher top body temperature (37.86 [0.89], 38.30 [0.90], and 38.21 [1.12]°C), higher respiratory rate (19.41 [2.23], 20.04 [2.93], and 22.13 [5.73] breaths per minute), a higher blood pH value (7.38 [0.06], 7.41 [0.06], and 7.43 [0.07]), a lower PaO2/FiO2 ratio (385.38 [38.08], 196.33 [94.35], and 109.00 [54.85]), higher demand of high-flow nasal cannula or higher-level oxygen support, and higher risk of fatality (0, 2 and 12 died) (all* P* for trend <0.05, Table 1). There was no difference in days from symptom onset to admission, proportion of sex, hypertension, diabetes mellitus, or presentation of clinical symptoms such as cough, expectoration, and fatigue across patients with different medical conditions (**Table 1**).

**Figure 2:**
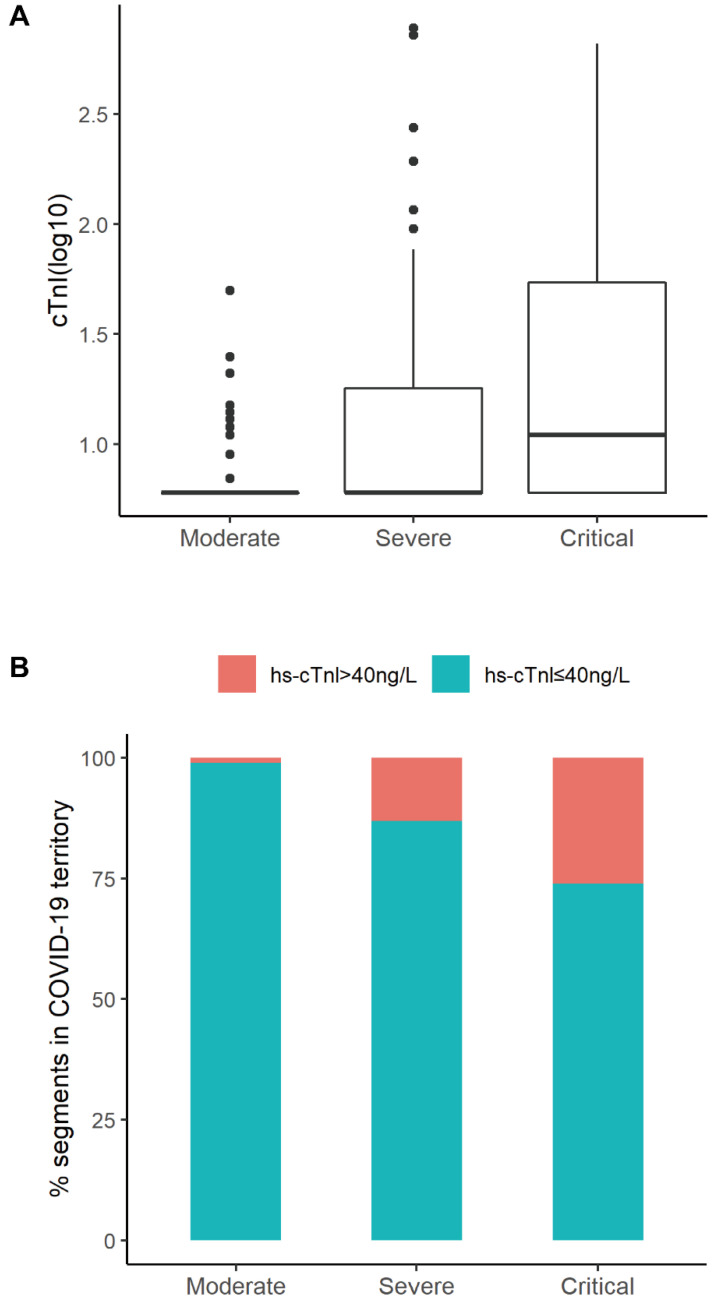
Distribution of hs-cTnI according to different severity of medical conditions. **A.** Distribution of cTnI (log) in subgroups (Moderate, Severe, Critical) of patients according to Clinical Classifications. **B.** Percentage of segments according to cTnI levels (cTnI ≤ 40ng/L or cTnI > 40ng/L ) in Moderate, Severe, or Critical group. The percentage of patients with elevated cTnI levels was positively correlated to the severity of disease conditions (Moderate 1.1%, Severe 13.1%, Critical 26.1%).

The hematological and biochemical parameters were evaluated and shown in **Table 2**. The severity of medical conditions were associated with decreased lymphocyte count (median [IQR]: 1.29 [0.91-1.79] ×109/L, 0.90 [0.68-1.25]×109/L, 0.68 [0.49-0.95]×109/L for those in moderate, severe, critical conditions, respectively), increased neutrophils (2.93 [2.29-4.24], 3.90 [2.33-5.96], and 6.22 [3.68-8.97]), increased procalcitonin (0.04 [0.03-0.05], 0.07 [0.04-0.14], and 0.16 [0.09-0.32] ng/mL), and elevated CRP levels (10.0 [5.0-37.2], 42.0 [8.8-83.1], and 101.5 [54.0-173.4] mg/L) (all* P* for trend<0.05, Table 2). The elevated levels of liver enzymes in the critically ill patients indicated liver injury occurring in these patients (Table 2). Levels of hs-cTnI increased gradually in patients with moderate (median [IQR]: 6.00 [6.00-6.00] ng/L), severe (6.00 [6.00-18.00]) and critical ill conditions (11.00 [6.00-56.75]), respectively (**Table 2 and Figure 2A**). In total, 27 (11.1%) patients had elevated hs-cTnI levels (>40 ng/L), including 1 patient with a moderate condition (1.1%), 14 with severe condition (13.1%), and 12 with critically ill condition (26.1%) (**Figure 2B**). Similarly, levels of other all cardiac indices, such as NT-proBNP, CK-MB, myoglobin, increased along with the increase in severity of condition (Table 2). The univariate regression results of significant clinical features with disease severity were presented in **Table S1**.

### Predictive performance of hs-cTnI for disease severity and 30-day fatality

**Figure 3:**
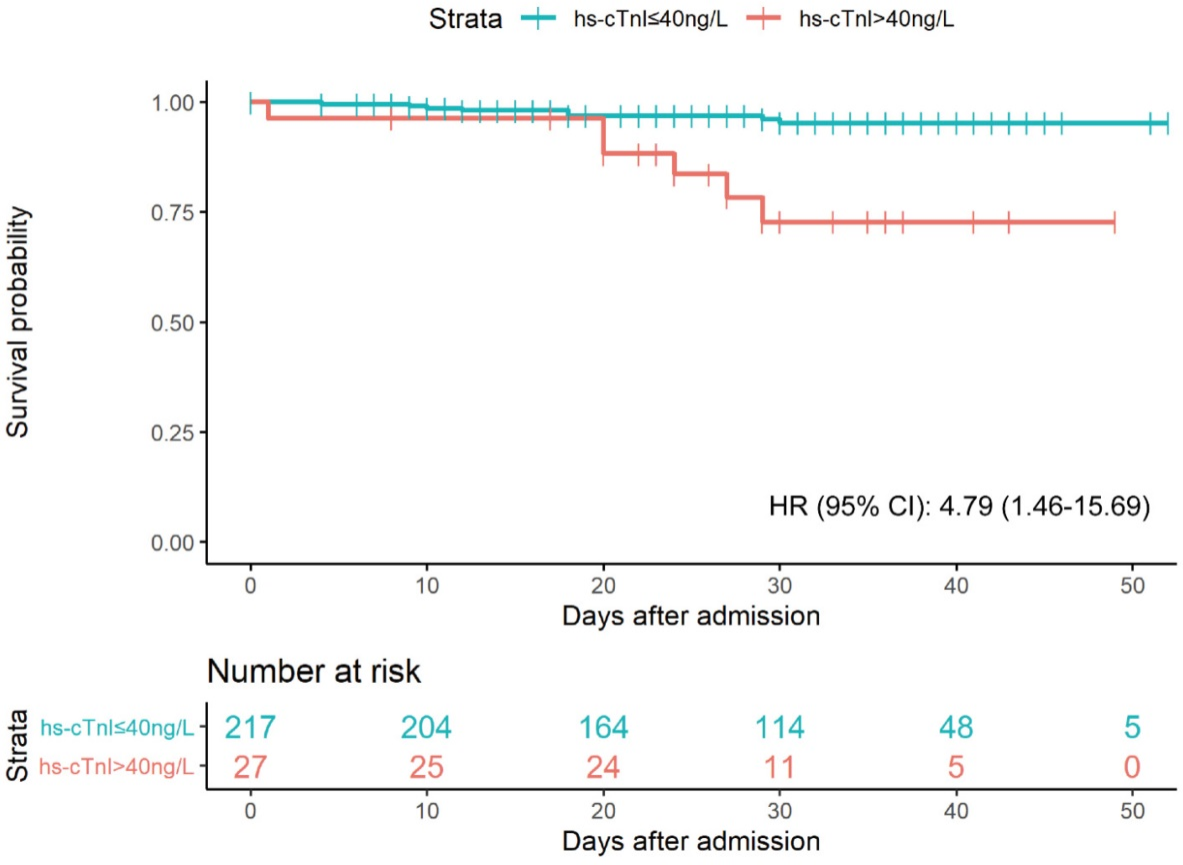
Kaplan-Meier plot for survial past hospital admission stratified by hs-cTnI levels. Patients were considered to be right-censored if they were discharged alive from hospital or were still in hospital at the time of data freeze (March 22, 2020). *P* were estimated from log-rank test. HR were estimated from multivariable Cox proportional hazards regression models after adjustment of age, sex, hypertension, diabetes, and eGFR.

As shown in **Figure 3**, a total of 14 patients died during the 7092 person-days of follow-up. 8 deaths (4%) occurred in the elevated hs-cTnI group (>40 ng/L), and 6 (22%) in the normal hs-cTnI group (≤40 ng/L). Patients with elevated hs-cTnI levels (>40 ng/L) were associated with higher fatality than those with a level of hs-cTnI in the normal range after controlling for age, sex, hypertension, diabetes, and eGFR (adjusted hazard ratio [95% CI]: 4.79 [1.46-15.69]). The univariate regression results of significant clinical features with 30-day fatality were presented in **Table S1**.

**Figure 4:**
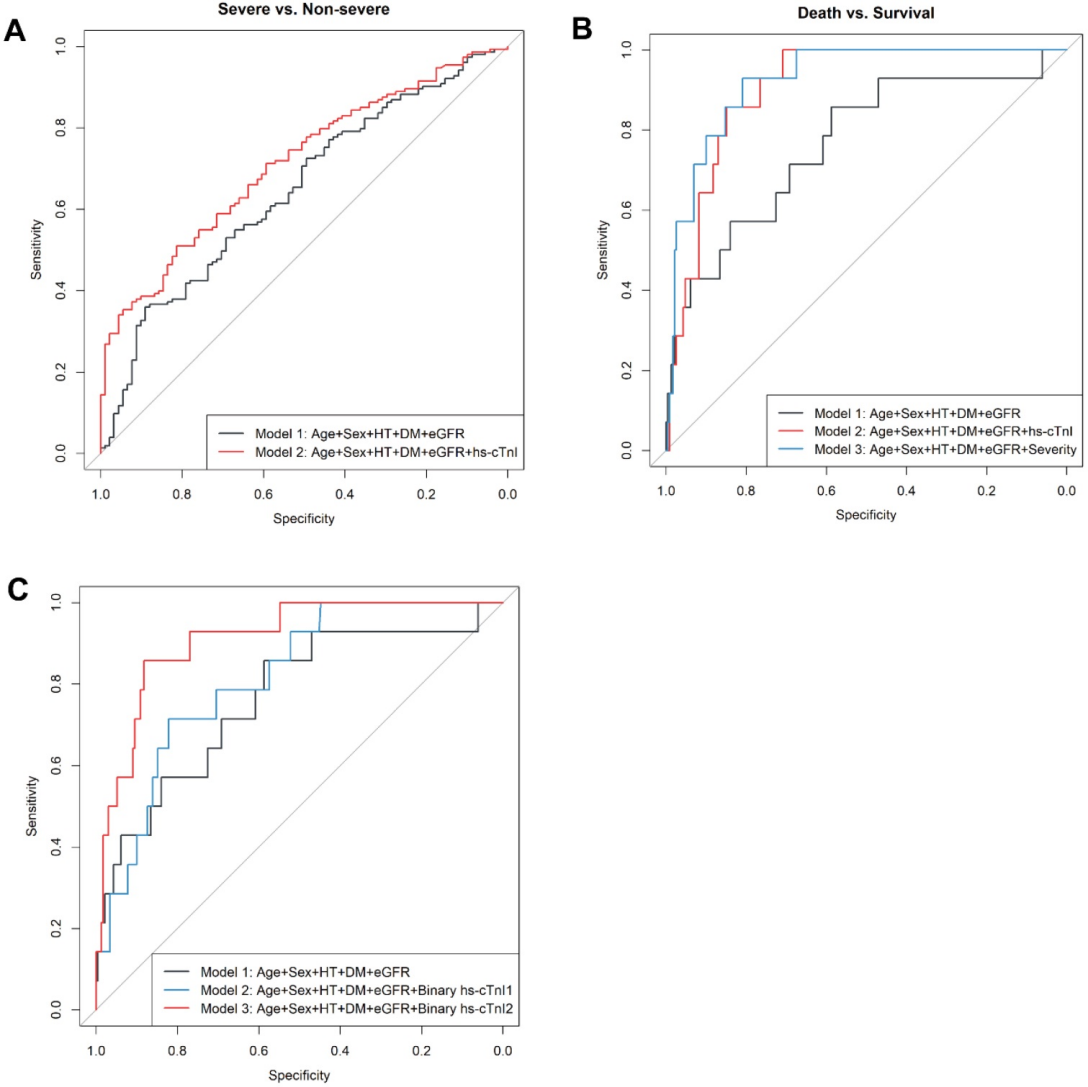
ROC curves of hierarchy predictive models. **A.** ROC curves for the classification of severe or worse medical conditions. Model 1 is the basic model, included age, sex, hypertension, diabetes, and eGFR, with an AUC1=0.645 (95% CI: 0.575-0.716); Model 2 further included hs-cTnI (log), with an AUC2 = 0.712 (95% CI: 0.648-0.776); and *P*=0.01 for difference between AUC 1 and AUC2. **B.** ROC curves for the prediction of in-hospital fatality. Model 1 is the basic model, included age, sex, hypertension, diabetes, and eGFR, with an AUC1 = 0.765 (95% CI: 0.621-0.908); Model 2: further included hs-cTnI (log), with an AUC2 =0.905 (95% CI: 0.853-0.957); and *P*=0.039 for difference between AUC 1 and AUC2. Model 3: Model 1 + clinical severity condition on admission (i.e., moderate, severe, critical), AUC3 = 0.925 (95% CI: 0.873-0.978); *P*=0.012 for difference between AUC1 and AUC3, and P=0.47 for difference between AUC2 and AUC3. **C.** ROC curves for the prediction of 30-day in-hospital fatality. Model 1 is the basic model, included age, sex, hypertension, diabetes, and eGFR, with AUC1 = 0.765 (95% CI: 0.621-0.908); Model 2: further included a binary hs-cTnI variable (cut point=40 ng/L), AUC2 = 0.815 (95% CI: 0.715-0.915); *P*=0.279 for difference between AUC1 and AUC2. Model 3: further included a binary hs-cTnI variable (cut point=20 ng/L), AUC3 = 0.911 (95% CI: 0.844-0.979); *P*=0.028 for difference between AUC1 and AUC3, and *P*=0.026 for difference between AUC2 and AUC3. Abbreviations: ROC, receiver operating characteristic; AUC, area under the curve; eGFR, estimated glomerular filtration rate; and hs-cTnI, high-sensitivity cardiac troponin I.

To specifically examine the prediction performance of cTnI levels on admission for prognosis of COVID19 patients, we built hierarchical prediction models using receiver-operating characteristic (ROC) curve analyses. Empirical clinical prognostic factors were included in the basic model (model 1), while the levels of cTnI on admission were further included in model 2. Compared with that in the basic model with empirical prognostic factors only (model 1), the performance for classification of a severe or critically ill medical condition of model 2 was significantly superior (AUC and 95% CI for the model 1: 0.645, 0.575-0.716; for the model 2: 0.712, 0.648-0.776; *P*=0.01 for difference) (**Figure 4A**). Meanwhile, compared with model 1, model 2 showed a borderline significant improved classification performance of a critically ill condition (AUC and 95% CI for model 1: 0.673, 0.592-0.754; for model 2: 0.725, 0.645-0.804; *P*=0.06 for difference) (**Figure S1**).

To predict 30-day in-hospital fatality, we developed the third model (model 3), in which both empirical prognostic factors and a condition severity variable on admission were included. The prediction performance of in-hospital fatality for both model 2 and model 3 were improved in comparison to model 1 (AUC and 95% CI for the model 1: 0.765, 0.621-0.908; the model 2: 0.905, 0.853-0.957; *P*=0.04 for the difference between AUC1 and AUC2; and for model 3: 0.925, 0.873-0.978; *P*=0.01 for the difference between AUC1 and AUC3) (**Figure 4B**). Levels of hs-cTnI on admission provided similar independent prediction performance of 30-day fatality compared to that of disease severity on admission (AUC and 95% CI: 0.905, 0.853-0.957; *P*=0.47 for the difference from that in model 3) (Figure 4B). In patients with available NT-proBNP measurements (N=200), the addition of NT-proBNP (log) to the basic model of 30-day fatality reached an AUC (95%CI) of 0.906 (0.844-0.968), which was not different from that in the model with basic factors and hs-cTnI (AUC: 0.905 [0.853-0.957],* P for difference*=0.49).

The ROC analysis indicated hs-cTnI as a potential sensitive biomarker for 30-day fatality (AUC and 95% CI: 0.877 [0.785-0.968]) (**Figure S2**). A threshold of 20.49 ng/L maximized the Youden index, providing a sensitivity of 0.857 and a specificity of 0.857 for 30-day fatality. This threshold also yielded a positive likelihood ratio of 5.97, and a negative likelihood ratio of 0.17. We further assessed the accuracy of adding a binary hs-cTnI variable with either our cutoff value (20.49 ng/L) or with the standard cut-off value (40.00 ng/L) to the basic model in prediction of 30-day fatality by respective AUCs. The prediction performance for the models with clinical prognostic factors plus a binary hs-cTnI variable (using either 20.49 ng/L or 40 ng/L as the cutoff point) was superior to that for the basic empirical model (AUC and 95% CI for the model with hs-cTnI cutoff point of 20.49 ng/L: 0.911 [0.844-0.979], *P* for its difference from AUC1 of the basic model=0.028; AUC and 95% CI for the model with hs-cTnI cutoff point of 40.00 ng/L: 0.815 [0.715-0.915], *P* for its difference from AUC1 of the basic model=0.279) (**Figure 4C**). Of note, using the selected cutoff point of 20.49 ng/L for hs-cTnI level provided better prediction performance of 30-day fatality compared with that using the clinical standard cutoff value of 40.00 ng/L (*P* for difference=0.026) (Figure 4C), suggesting the better clinical relevance of this new hs-cTnI cutoff value (20.49 ng/L).

### Correlation between myocardial injury and other clinical features

A total of 45 patients (18%) had myocardial injury using the above-mentioned selected cutoff value of hs-cTnI levels (≥20.49 ng/L). Compared with patients with no myocardial injury on admission, those with myocardial injury were more likely to be older (mean [SD]: 70.84 [12.06] vs. 60.71 [13.04] years), had lower levels of PaO2/FiO2 ratio (242.53 [123.39] vs. 140.50 [94.44] mmHg) and higher demand of high-flow nasal cannula or higher-level oxygen support (**Table S2**). In addition, those patients had lower counts of lymphocyte, CD4+ and CD8+ T cells and elevated levels of CRP, procalcitonin, other myocardial indicators, and liver enzymes (**Table S3**).

## Discussion

In the current study, we investigated clinical characteristics, particularly the cardiac biomarker hs-cTnI, in 244 COVID-19 patients without prior history of cardiovascular disease or renal dysfunction. Our study suggested that incorporation of hs-cTnI levels on admission remarkably improved in fatality prediction of the first 30 days since admission over an empirical risk prediction model. In addition, an optimal cutoff value of 20.49 ng/L for hs-cTnI was chosen based on AUC analysis. The newly selected 20.49 ng/L of hs-cTnI predicted 30-day fatality with both specificity and sensitivity values of 85.7%, provided better prediction performance than that in the standard cutoff value of 40 ng/L.

### Myocardial injury in COVID-19, and possible mechanisms

Although the exact pathophysiological mechanism of myocardial injury due to SARS-CoV-2 infection has not been fully elucidated, evidence has suggested that it may involve direct virus infection of myocardial tissue, and indirect pathways such as myocardial infarction, immune dysregulation, inflammation and hypoxia 10, 21.

The pathophysiology of SARS-CoV-2 infection was similar to that in other coronavirus infections, such as SARS-associated coronavirus (SARS-CoV) and Middle East Respiratory Syndrome Coronavirus (MERS-CoV) 22-24. It has been reported that SARS-CoV viral RNA was detected in autopsied human heart tissue 23, thus direct cardiac involvement in COVID-19 would be highly expected. Coronavirus entries into host cells by binding its spike-like capsid to the metallopeptidase, Angiotensin Converting Enzyme-2 (ACE-2) in the host cells 10, suggested the possibility of direct infection of SARS-CoV-2 in myocardial cells. Although earlier autopsy results did not find SARS-CoV-2 genome or substantial damage in the heart tissue except for a few interstitial mononuclear inflammatory infiltration 25, 26, the most recent autopsy reported detectable SARS-CoV-2 RNA in autopsied heart in consecutive COVID-19 patients 27. Once SARS-CoV-2 directly attacks the myocardial tissue, the biggest concern is that susceptible individuals may go on to develop chronic myocarditis and dilated cardiomyopathy. In addition, ACE-2 is also localized in other organs (e.g., lung, intestinal epithelium, vascular endothelium, and kidneys) 10, 28. The ACE-2 suppression by SARS-CoV-2 will result in the angiotensin II/angiotensin 1-7 imbalance, which would cause further injuries to the heart in COVID-19 patients 29.

Acute respiratory infections, such as influenza and viral pneumonia 30, 31, have been associated with short-term risk of myocardial infarction 32-34, and this may apply to SARS-CoV-2 infections. It has been postulated that increased systematic and intraplaque inflammatory activity, persistent destabilization of the plaques, and the prothrombotic and procoagulant state may collectively cause myocardial infarction in these acute respiratory infections 35. Acute infection triggered myocardial infarction has been observed in COVID-19 patients, especially in severe patients 18, 36-38. Shi found that 14 out of 416 patients (3.36%) may have developed myocardial ischemia, with features consistent with ST-elevation myocardial infarction 18. In this cohort, we did not observe patients with typical features of ST-elevation myocardial infarction, which may be partly due to the exclusion criteria (preexisting cardiovascular disease). However, microinfarction caused by the prothrombotic and procoagulant state could be a possibility.

Consistent with previous studies 5, 37, 39, patients with myocardial injury had elevated levels of hs-CRP and lower levels of lymphocytes, CD4^+^ and CD8^+^ T cells, compared to those without myocardial injury, suggesting myocardial injury may predispose COVID-19 patients to poor prognosis, partially through increased systemic inflammation and dysregulation of the immune system 40. In addition, patients with myocardial injury were more likely to have a higher respiratory frequency and a lower PaO2/FiO2 ratio, suggesting that hypoxia might be one cause of myocardial injury.

### Prognostic value of hs-cTnI and its relation of clinical features

In our study, the prevalence of myocardial injury was 18% determined by the selected hs-cTnI cutoff value of 20.49 ng/L or 11% by the clinical standard cutoff value of 40ng/L. In either case, the prevalence of myocardial injury identified in this study was lower than that in recent reports in COVID-19 patients 41-44 , which could be explained by the exclusion of COVID-19 patients with pre-existing cardiovascular disease and renal dysfunction in our analysis. In addition, our study suggested that patients with elevated hs-cTnI were more likely to be older, had lower lymphocyte counts and elevated levels of inflammation index, procalcitonin, and liver enzymes. Among these patients with no underlying cardiovascular disease and renal dysfunction, the elevated hs-cTnI level of >40 ng/L was related with a four-fold increased fatality risk independent of empirical risk factors. This finding was in line with a recent report showing myocardial injury was associated with an adjusted hazard ratio of 4.56 (95% CI: 1.28, 16.28) for fatality in COVID-19 patients 45.

For the first time, our study elicited a new threshold of elevated hs-cTnI levels (20.49 ng/L) which was about half of the upper reference limit (the standard cutoff point, i.e., 40 ng/L). We believe this new threshold of hs-cTnI levels could provide not only better independent prediction performance of fatality compared with the standard cutoff point, but also comparable independent prediction performance of fatality compared with the clinical severity classification. Our data underscored the importance of hs-cTnI in risk stratification and patient classification, and further echoed recent findings in which highlighted the role of hs-cTnI in suspected myocardial infarction 46 and heart failure 47.

## Limitations

Nevertheless, this study is subject to several limitations. First, most COVID-19 patients did not have dynamic measurements of hs-cTnI levels during their stay in hospital. Therefore, we could not track the peak value of hs-cTnI during the disease course, which would be helpful to identify the timeline in which myocardial damage occurred and sustained. Second, we did not collect information of body mass index or smoking history, which may be important predictors to fatality in COVID-19 patients. Third, our hospital did not admit COVID-19 patients with mild conditions during the study period, thus our findings may not be generalizable to those patients. Last but not least, the clinical information was available for only 30 days in our study. The long-term prognosis would certainly provide additional valuable findings.

## Conclusion

Our study showed that 11% of COVID-19 patients had elevated hs-cTnI levels by the standard cutoff value of 40 ng/L, even they did not have preexisting cardiovascular disease or renal dysfunction. The hs-cTnI level on admission provided additional prediction of 30-day fatality in COVID-19 patients over the empirical clinical prognostic factors. The added prediction performance by this single, simple, and quick index of hs-cTnI level was comparable to that by the complicated clinical severity classification. Furthermore, our study demonstrated the selected cutoff hs-cTnI level of 20.49 ng/L provided better prediction performance than that using the standard cutoff point. These observations may provide strong evidence of a simple and efficient predictor of fatality in COVID-19 patients and provide critical supporting data to make a timely clinical decision.

## Supplementary Material

Supplementary figures and tables.

## Figures and Tables

**Table 1:** Clinical presentations and features by medical conditions in 244 patients with COVID-19

	Moderate (n=91)	Severe (n=107)	Critical (n=46)	*P*_trend_
Age (years)	59.79 ± 13.49	62.20 ± 13.43	68.98 ± 11.26	<0.001
Time since symptom onset (days)	11.27 ± 4.81	11.64 ± 5.48	10.91 ± 4.69	0.83
Male Sex, No. (%)	44 (48.35)	63 (58.88)	26 (56.52)	0.25
**Coexisting conditions**				
Hypertension, No. (%)	21 (23.08)	41 (38.32)	13 (28.26)	0.27
Diabetes Mellitus, No. (%)	12 (13.19)	14 (13.08)	10 (21.74)	0.25
Highest temperature (°C)	37.86 ± 0.89	38.30 ± 0.90	38.21 ± 1.12	0.01
Heart rate (beat per minute)	87.26 ± 14.48	87.08 ± 12.75	91.85 ± 18.17	0.14
Respiration rate (breath per minute)	19.41 ± 2.23	20.04 ± 2.93	22.13 ± 5.73	<0.001
Mean arterial pressure (mmHg)	94.01 ± 11.72	94.95 ± 10.65	94.72 ± 11.61	0.65
pH	7.38 ± 0.07	7.41 ± 0.06	7.43 ± 0.07	0.002
PaO_2_ (mmHg)	77.20 ± 7.81	68.33 ± 14.89	59.51 ± 12.39	<0.001
PaCO_2_ (mmHg)	43.57 ± 7.06	40.77 ± 7.48	37.67 ± 5.71	<0.001
PaO_2_/FiO_2_ ratio	385.38 ± 38.08	196.33 ± 94.35	109.00 ± 54.85	<0.001
**Signs and symptoms, No. (%)**				
Cough	55 (60.44)	73 (68.22)	31 (67.39)	0.33
Expectoration	9 (9.89)	24 (22.43)	11 (23.91)	0.02
Shortness of breath	24 (26.37)	40 (37.38)	24 (52.17)	0.003
Chest pain	2 (2.20)	1 (0.93)	0 (0.00)	0.25
Rhinorrhoea	1 (1.10)	2 (1.87)	0 (0.00)	0.72
Diarrhoea	10 (10.99)	12 (11.21)	8 (17.39)	0.35
Muscle ache	10 (10.99)	6 (5.61)	5 (10.87)	0.72
Fatigue	31 (34.07)	36 (33.64)	16 (34.78)	0.95
Anorexia	14 (15.38)	23 (21.50)	8 (17.39)	0.60
Death	0 (0.00)	2 (1.87)	12 (26.09)	<0.001
**Oxygen support, No. (%)**				<0.001
Non-inhalation oxygen therapy	73 (80.22)	13 (13.27)	0 (0.00)	
Nasal cannula	18 (19.78)	60 (61.22)	7 (15.56)	
Non-invasive ventilation or high-flow nasal cannula	0 (0.00)	23 (23.47)	23 (51.11)	
Invasive mechanical ventilation	0 (0.00)	2 (2.04)	14 (31.11)	
Extracorporeal membrane oxygenation	0 (0.00)	0 (0.00)	1 (2.22)	

Values were presented as ^a^ median (interquartile range) for continuous variables with a skewed distribution, ^b^ mean± standard deviation for continuous variables with a normal distribution, or No. (%) for categorical variables. *P* for trend was calculated from the linear or logistic regression model. Abbreviations: pH, potential of Hydrogen; pO2, partial pressure of oxygen in blood; pCO2, partial pressure of carbon dioxide in blood.

**Table 2:** Blood biochemical features by clinical conditions in 244 patients with COVID-19

	Moderate (n=91)	Severe (n=107)	Critical (n=46)	*P*_trend_	Normal range
White blood cell count (×10^9^/L)^a^	5.22 (3.92 - 6.60)	5.72 (4.01 - 7.34)	7.85 (4.62 - 10.61)	0.006	3.5-9.5
Lymphocyte count (×10^9^/L)^a^	1.29 (0.91 - 1.79)	0.90 (0.68 - 1.25)	0.68 (0.49 - 0.95)	<0.001	1.1-3.2
Neutrophil count (×10^9^/L)^a^	2.93 (2.29 - 4.24)	3.90 (2.33 - 5.96)	6.22 (3.68 - 8.97)	<0.001	1.8-6.3
CD4 (/μL)^a^	510.00 (343.00 - 691.00)	378.00 (234.00 - 518.00)	210.50 (159.25 - 349.00)	<0.001	404-1612
CD8 (/μL)^a^	303.00 (203.00 - 453.00)	194.00 (120.00 - 273.00)	119.50 (68.50 - 160.25)	<0.001	220-1129
Haemoglobin (g/L)^b^	124.92 ± 13.53	124.45 ± 16.45	129.17 ± 15.75	0.17	130-175
Platelet count (×10^9^/L)^a^:	247.00 (189.00 - 299.50)	209.00 (157.00 - 274.00)	221.00 (174.75 - 254.75)	0.03	100-300
<100, No. (%)	1 (1.10)	6 (5.61)	4 (8.70)	0.09	
≥100, No. (%)	90 (98.90)	101 (94.39)	42 (91.30)		
C-Reactive Protein (mg/L)^a^	10.00 (5.00 - 37.15)	42.00 (8.75 - 83.10)	101.45 (54.00 - 173.43)	<0.001	0-10
Procalcitonin (ng/mL)^a^:	0.04 (0.03 - 0.05)	0.07 (0.04 - 0.14)	0.16 (0.09 - 0.32)	<0.001	<0.1
<0.1, No. (%)	78 (85.71)	69 (64.49)	13 (28.26)	<0.001	
≥0.1, No. (%)	13 (14.29)	38 (35.51)	33 (71.74)		
CK-MB (ng/ml)^a^	0.82 (0.61 - 1.25)	1.03 (0.73 - 1.56)	1.58 (0.88 - 2.59)	0.007	0-5
Hs-cTnI (ng/L)^a^	6.00 (6.00 - 6.00)	6.00 (6.00 - 18.00)	11.00 (6.00 - 56.75)	<0.001	0.04
≤40, No. (%)	90 (98.90)	93 (86.92)	34 (73.91)	<0.001	
>40, No. (%)	1 (1.10)	14 (13.08)	12 (26.09)		
Myoglobin (μg/L)	34.00 (23.38 - 51.41)	39.35 (29.21 - 74.19)	66.37 (43.18 - 109.50)	0.006	0-110
NTpro-BNP (pg/ml)	67.00 (26.14 - 176.50)	161.80 (64.52 - 355.18)	465.00 (195.25 - 993.50)	<0.001	0-450
eGFR (ml/min/1.73 m^2^)^a^	96.00 (90.84 - 105.31)	94.77 (86.63 - 105.72)	93.31 (78.50 - 101.20)	0.25	> 60
Alanine aminotransferase (U/L)^a^	23.00 (15.00 - 41.00)	29.00 (19.00 - 47.50)	28.50 (20.25 - 48.75)	0.02	9-50
Aspartate aminotransferase (U/L)^a^	23.00 (18.00 - 36.00)	33.00 (22.00 - 45.50)	42.00 (31.25 - 53.00)	<0.001	15-40
≤ 40 U/L, No. (%)	74 (81.32)	73 (68.22)	22 (47.83)	<0.001	
>40 U/L, No. (%)	17 (18.68)	34 (31.78)	24 (52.17)		
Alkaline phosphatase (U/L)^a^	61.00 (51.50 - 72.00)	65.00 (52.00 - 82.00)	67.00 (51.00 - 102.25)	0.34	45-125
Gamma glutamyl transpeptidase (U/L)^a^	27.00 (15.50 - 45.00)	31.00 (21.50 - 60.00)	39.50 (25.00 - 83.00)	<0.001	10-60
Total bilirubin (μmol/L)^a^	10.40 (8.05 - 14.00)	11.20 (8.45 - 14.85)	13.80 (9.25 - 17.20)	0.07	0-23
Conjugated bilirubin (μmol/L)^a^	3.50 (2.80 - 5.00)	4.20 (3.15 - 5.55)	5.45 (3.80 - 7.38)	0.009	0-8
Serum creatinine (μmol/L)^a^	60.00 (51.00 - 70.00)	64.00 (53.50 - 75.00)	65.00 (48.25 - 73.75)	0.79	57-97
Random blood glucose (mmol/L)^a^	5.40 (4.70 - 6.85)	5.60 (4.90 - 6.80)	7.00 (5.53 - 9.43)	0.01	<11.1
Potassium (mmol/L)^a^	4.00 (3.64 - 4.32)	3.98 (3.66 - 4.45)	3.88 (3.47 - 4.36)	0.83	3.5-5.3
Sodium (mmol/L)^a^	142.00 (139.00 - 144.00)	141.00 (137.50 - 144.00)	138.00 (136.00 - 142.75)	0.13	137-147
Triglyceride (mmol/L)^a^	1.24 (0.92 - 1.68)	1.23 (0.99 - 1.65)	1.25 (0.97 - 1.95)	0.37	5.2
Total cholesterol (mmol/L)^a^	4.12 (3.32 - 4.58)	3.79 (3.29 - 4.51)	3.71 (3.18 - 4.08)	0.03	1.7
Creatine kinase (U/L)^a^	52.00 (37.50 - 88.00)	63.00 (39.50 - 105.50)	69.50 (37.00 - 144.50)	0.12	50-310
Lactate dehydrogenase (U/L)^a^	217.00 (182.50 - 275.50)	317.00 (244.50 - 422.00)	434.50 (357.00 - 538.50)	<0.001	120-250
≤250, No. (%)	56 (61.54)	30 (28.04)	5 (10.87)	<0.001	
>250, No. (%)	35 (38.46)	77 (71.96)	41 (89.13)		

Values were presented as ^a^ median (interquartile range) for continuous variables with a skewed distribution, ^b^ mean± standard deviation for continuous variables with a normal distribution, or No. (%) for categorical variables. *P* for trend was calculated from the linear or logistic regression model after adjustment of age and sex. Abbreviations: hs-cTnI, high-sensitivity cardiac troponin I; NT-proBNP, pro-B-type natriuretic peptide; and CK-MB, creatinine kinase.

## References

[1] World Health Organization Coronavirus disease (COVID-19) pandemic. 2020.

[2] Novel Coronavirus Pneumonia Emergency Response Epidemiology Team (2020). The epidemiological characteristics of an outbreak of 2019 novel coronavirus diseases (COVID-19) in China. Zhonghua Liu Xing Bing Xue Za Zhi.

[3] Chen N, Zhou M, Dong X, Qu J, Gong F, Han Y (2020). Epidemiological and clinical characteristics of 99 cases of 2019 novel coronavirus pneumonia in Wuhan, China: a descriptive study. Lancet.

[4] Huang C, Wang Y, Li X, Ren L, Zhao J, Hu Y (2020). Clinical features of patients infected with 2019 novel coronavirus in Wuhan, China. Lancet.

[5] Aggarwal G, Cheruiyot I, Aggarwal S, Wong J, Lippi G, Lavie CJ (2020). Association of cardiovascular disease with coronavirus disease 2019 (COVID-19) severity: a meta-analysis. Current Problems in Cardiology.

[6] Gunsolus I, Sandoval Y, Smith SW, Sexter A, Schulz K, Herzog CA (2018). Renal Dysfunction Influences the Diagnostic and Prognostic Performance of High-Sensitivity Cardiac Troponin I. J Am Soc Nephrol.

[7] Liu Y, Yang Y, Zhang C, Huang F, Wang F, Yuan J (2020). Clinical and biochemical indexes from 2019-nCoV infected patients linked to viral loads and lung injury. Sci China Life Sci.

[8] Li L, Yang L, Gui S, Pan F, Ye T, Liang B (2020). Association of clinical and radiographic findings with the outcomes of 93 patients with COVID-19 in Wuhan, China. Theranostics.

[9] Zheng YY, Ma YT, Zhang JY, Xie X (2020). COVID-19 and the cardiovascular system. Nat Rev Cardiol.

[10] Hamming I, Timens W, Bulthuis ML, Lely AT, Navis G, van Goor H (2004). Tissue distribution of ACE2 protein, the functional receptor for SARS coronavirus. A first step in understanding SARS pathogenesis. J Pathol.

[11] Turner AJ, Hiscox JA, Hooper NM (2004). ACE2: from vasopeptidase to SARS virus receptor. Trends in pharmacological sciences.

[12] Mehra MR, Desai SS, Kuy S, Henry TD, Patel AN (2020). Cardiovascular Disease, Drug Therapy, and Mortality in Covid-19. N Engl J Med.

[13] Cremer PC (2020). SARS-CoV-2 and myocardial injury: Few answers, many questions. Cleve Clin J Med.

[14] WHO Clinical management of severe acute respiratory infection when Novel coronavirus (nCoV) infection is suspected: interim guidance. 2020.

[15] Wu C, Chen X, Cai Y, Xia J, Zhou X, Xu S (2020). Risk Factors Associated With Acute Respiratory Distress Syndrome and Death in Patients With Coronavirus Disease 2019 Pneumonia in Wuhan, China. JAMA Intern Med.

[16] Lighter J, Phillips M, Hochman S, Sterling S, Johnson D, Francois F (2020). Obesity in patients younger than 60 years is a risk factor for Covid-19 hospital admission. Clin Infect Dis.

[17] Zhou F, Yu T, Du R, Fan G, Liu Y, Liu Z (2020). Clinical course and risk factors for mortality of adult inpatients with COVID-19 in Wuhan, China: a retrospective cohort study. Lancet.

[18] Chen C, Yan J, Zhou N, Zhao J, Wang D (2020). Analysis of myocardial injury in patients with COVID-19 and association between concomitant cardiovascular diseases and severity of COVID-19. Zhonghua xin xue guan bing za zhi.

[19] Chen T, Wu D, Chen H, Yan W, Yang D, Chen G (2020). Clinical characteristics of 113 deceased patients with coronavirus disease 2019: retrospective study. Bmj.

[20] Krzanowski WJ, Hand DJ ROC curves for continuous data: Crc Press. 2009.

[21] Babapoor-Farrokhran S, Gill D, Walker J, Rasekhi RT, Bozorgnia B, Amanullah A Myocardial injury and COVID-19: Possible mechanisms. Life Sciences. 2020: 117723.

[22] Alhogbani T (2016). Acute myocarditis associated with novel Middle east respiratory syndrome coronavirus. Ann Saudi Med.

[23] Oudit GY, Kassiri Z, Jiang C, Liu PP, Poutanen SM, Penninger JM (2009). SARS-coronavirus modulation of myocardial ACE2 expression and inflammation in patients with SARS. Eur J Clin Invest.

[24] Inciardi RM, Lupi L, Zaccone G, Italia L, Raffo M, Tomasoni D (2020). Cardiac Involvement in a Patient With Coronavirus Disease 2019 (COVID-19). JAMA Cardiol.

[25] Wichmann D, Sperhake JP, Lutgehetmann M, Steurer S, Edler C, Heinemann A (2020). Autopsy Findings and Venous Thromboembolism in Patients With COVID-19. Ann Intern Med.

[26] Fox SE, Akmatbekov A, Harbert JL, Li G, Quincy Brown J, Vander Heide RS (2020). Pulmonary and cardiac pathology in African American patients with COVID-19: an autopsy series from New Orleans. Lancet Respir Med.

[27] Wichmann D, Sperhake J-P, Lütgehetmann M, Steurer S, Edler C, Heinemann A (2020). Autopsy findings and venous thromboembolism in patients with COVID-19: a prospective cohort study. Annals of internal medicine.

[28] Tikellis C, Bernardi S, Burns WC (2011). Angiotensin-converting enzyme 2 is a key modulator of the renin-angiotensin system in cardiovascular and renal disease. Curr Opin Nephrol Hypertens.

[29] Tersalvi G, Vicenzi M, Calabretta D, Biasco L, Pedrazzini G, Winterton D (2020). Elevated Troponin in Patients With Coronavirus Disease 2019: Possible Mechanisms. J Card Fail.

[30] Kwong JC, Schwartz KL, Campitelli MA, Chung H, Crowcroft NS, Karnauchow T (2018). Acute Myocardial Infarction after Laboratory-Confirmed Influenza Infection. N Engl J Med.

[31] Warren-Gash C, Geretti AM, Hamilton G, Rakhit RD, Smeeth L, Hayward AC (2013). Influenza-like illness in acute myocardial infarction patients during the winter wave of the influenza A H1N1 pandemic in London: a case-control study. BMJ Open.

[32] Clayton TC, Thompson M, Meade TW (2008). Recent respiratory infection and risk of cardiovascular disease: case-control study through a general practice database. Eur Heart J.

[33] Warren-Gash C, Blackburn R, Whitaker H, McMenamin J, Hayward AC (2018). Laboratory-confirmed respiratory infections as triggers for acute myocardial infarction and stroke: a self-controlled case series analysis of national linked datasets from Scotland. Eur Respir J.

[34] Corrales-Medina VF, Alvarez KN, Weissfeld LA, Angus DC, Chirinos JA, Chang CC (2015). Association between hospitalization for pneumonia and subsequent risk of cardiovascular disease. JAMA.

[35] Musher DM, Abers MS, Corrales-Medina VF (2019). Acute Infection and Myocardial Infarction. N Engl J Med.

[36] Hinterseer M, Zens M, Wimmer RJ, Delladio S, Lederle S, Kupatt C (2020). Acute myocardial infarction due to coronary stent thrombosis in a symptomatic COVID-19 patient. Clin Res Cardiol.

[37] Schiavone M, Gobbi C, Biondi-Zoccai G, D'Ascenzo F, Palazzuoli A, Gasperetti A (2020). Acute Coronary Syndromes and Covid-19: Exploring the Uncertainties. J Clin Med.

[38] Sgura FA, Arrotti S, Cappello CG, Boriani G (2020). Complicated myocardial infarction in a 99-year-old lady in the era of COVID-19 pandemic: from the need to rule out coronavirus infection to emergency percutaneous coronary angioplasty. Intern Emerg Med.

[39] Xu PP, Tian RH, Luo S, Zu ZY, Fan B, Wang XM (2020). Risk factors for adverse clinical outcomes with COVID-19 in China: a multicenter, retrospective, observational study. Theranostics.

[40] Tersalvi G, Vicenzi M, Calabretta D, Biasco L, Pedrazzini G, Winterton D (2020). Elevated Troponin in Patients With Coronavirus Disease 2019: Possible Mechanisms. Journal of cardiac failure.

[41] Deng Q, Hu B, Zhang Y, Wang H, Zhou X, Hu W (2020). Suspected myocardial injury in patients with COVID-19: Evidence from front-line clinical observation in Wuhan, China. Int J Cardiol.

[42] Guo T, Fan Y, Chen M, Wu X, Zhang L, He T (2020). Cardiovascular Implications of Fatal Outcomes of Patients With Coronavirus Disease 2019 (COVID-19). JAMA Cardiol.

[43] Xie Y, You Q, Wu C, Cao S, Qu G, Yan X (2020). Impact of Cardiovascular Disease on Clinical Characteristics and Outcomes of Coronavirus Disease 2019 (COVID-19). Circ J.

[44] Aggarwal S, Garcia-Telles N, Aggarwal G, Lavie C, Lippi G, Henry BM (2020). Clinical features, laboratory characteristics, and outcomes of patients hospitalized with coronavirus disease 2019 (COVID-19): Early report from the United States. Diagnosis (Berl).

[45] Shi S, Qin M, Cai Y, Liu T, Shen B, Yang F (2020). Characteristics and clinical significance of myocardial injury in patients with severe coronavirus disease 2019. Eur Heart J.

[46] Neumann JT, Twerenbold R, Ojeda F, Sörensen NA, Chapman AR, Shah AS (2019). Application of high-sensitivity troponin in suspected myocardial infarction. New England Journal of Medicine.

[47] Myhre PL, Claggett B, Ballantyne CM, Selvin E, Røsjø H, Omland T (2019). Association between circulating Troponin concentrations, left ventricular systolic and diastolic functions, and incident heart failure in older adults. JAMA cardiology.

