# Supplementary material for: Myocardial injury and COVID-19: Serum hs-cTnI level in risk stratification and the prediction of 30-day fatality in COVID-19 patients with no prior cardiovascular disease
Source: Theranostics. 2020 Jul 29;10(21):9663–73. doi: 10.7150/thno.47980 (PMC7449913; doi:10.7150/thno.47980)
Supplement: Supplementary file 1 — Supplementary figures and tables. [file thnov10p9663s1.pdf]

Table S1. Univariate analysis with condition severity and 30-day fatality

Variables	Critical vs Non-critical		Severe vs Non-severe		Death vs Survival	
	**Beta ± SE**	***P* value**	**Beta ± SE**	***P* value**	**Beta ± SE**	***P* value**
Age (years)	0.05 ± 0.02	0.001	0.02 ± 0.01	0.01	0.09 ± 0.03	0.004
Sex (males vs females)	-0.10 ± 0.33	0.76	-0.40 ± 0.27	0.14	-0.11 ± 0.56	0.84
Hypertension (yes vs no)	-0.15 ± 0.36	0.69	0.60 ± 0.30	0.047	-0.11 ± 0.61	0.86
Diabetes (yes vs no)	0.61 ± 0.41	0.14	0.20 ± 0.38	0.60	0.91 ± 0.62	0.14
eGFR (ml/min/1.73 m^2)*	-2.68 ± 1.47	0.07	-3.92 ± 1.61	0.01	-2.30 ± 2.29	0.31
White blood cell count (×10^9/L)*	3.46 ± 0.92	<0.001	1.07 ± 0.69	0.12	0.37 ± 1.41	0.79
Lymphocyte count (×10^9/L)*	-4.07 ± 0.87	<0.001	-4.63 ± 0.77	<0.001	-4.96 ± 1.41	<0.001
Neutrophil count (×10^9/L)*	3.14 ± 0.71	<0.001	1.73 ± 0.53	0.001	0.26 ± 1.03	0.80
C-Reactive Protein (mg/L)*	2.55 ± 0.46	<0.001	1.61 ± 0.27	<0.001	2.98 ± 0.90	<0.001
Hs-cTnl (ng/L)*	1.36 ± 0.33	<0.001	2.77 ± 0.66	<0.001	2.10 ± 0.46	<0.001
NT-proBNP (pg/ml)*	1.92 ± 0.38	<0.001	1.32 ± 0.28	<0.001	2.86 ± 0.69	<0.001
Aspartate aminotransferase (U/L)*	2.51 ± 0.73	<0.001	2.84 ± 0.66	<0.001	2.30 ± 1.15	0.046
Alkaline phosphatase (U/L)*	0.75 ± 0.85	0.38	0.96 ± 0.67	0.15	2.76 ± 1.42	0.052
Binary hs-cTnI1 (>40ng/L vs ≤40ng/L)	1.46 ± 0.43	0.001	2.91 ± 1.03	0.005	2.01 ± 0.59	<0.001
Binary hs-cTnI2 (>20ng/L vs ≤20ng/L)	1.54 ± 0.37	<0.001	2.41 ± 0.61	<0.001	3.58 ± 0.79	<0.001

*Variables were log$_{10}$ transformed before analysis.

Beta, SE, and *P* value were estimated from logistic regression models.

SE, standard error; NT-proBNP, N-terminal pro-B-type natriuretic peptide.

Table S2. hs-cTnI levels and characteristics of 244 patients with COVID-19

	hs-cTnI normal	hs-cTnI elevated	*P* value
	(N=199, hs-cTnI≤20.49 ng/L)	(N=45, hs-cTnI＞20.49 ng/L)	
Age (years)	60.71 ± 13.04	70.84 ± 12.06	< 0.001
Time since symptom onset (days)	11.42 ± 5.07	11.11 ± 5.22	0.66
Male Sex, No.(%)	105 (52.76)	28 (62.22)	0.32
**Coexisting conditions**			
Hypertension, No.(%)	61 (30.65)	14 (31.11)	1
Diabetes Mellitus, No.(%)	28 (14.07)	8 (17.78)	0.69
Highest temperature (℃)	38.12 ± 0.96	38.13 ± 0.95	0.84
Heart rate (beat per minute)	87.31 ± 13.81	91.31 ± 17.45	0.27
Respiration rate (breath per minute)	19.92 ± 2.95	21.40 ± 5.35	0.38
Mean arterial pressure (mmHg)	94.45 ± 10.49	95.02 ± 14.05	0.98
pH	7.40 ± 0.07	7.43 ± 0.07	0.037
PaO2 (mmHg)	70.72 ± 13.02	59.92 ± 15.06	< 0.001
PaCO2 (mmHg)	41.77 ± 7.33	37.00 ± 5.55	< 0.001
PaO_2/FiO_2 ratio	242.53 ± 123.39	140.50 ± 94.44	< 0.001
**Signs and symptoms, No.(%)**			
Cough	129 (64.82)	30 (66.67)	0.95
Expectoration	38 (19.10)	6 (13.33)	0.49
Shortness of breath	70 (35.18)	18 (40.00)	0.66
Chest pain	3 (1.51)	0 (0.00)	1
Rhinorrhoea	3 (1.51)	0 (0.00)	1
Diarrhoea	22 (11.06)	8 (17.78)	0.32
Muscle ache	21 (10.55)	0 (0.00)	0.02
Fatigue	65 (32.66)	18 (40.00)	0.44
Anorexia	35 (17.59)	10 (22.22)	0.61
Death	2 (1.01)	12 (26.67)	< 0.001
**Oxygen support, No.(%)**			< 0.001
Non-inhalation oxygen therapy	81 (41.33)	5 (13.16)	
Nasal cannula	69 (35.20)	16 (42.11)	
Non-invasive ventilation or high-flow nasal cannula	39 (19.90)	7 (18.42)	
Invasive mechanical ventilation	7 (3.57)	9 (23.68)	
Extracorporeal membrane oxygenation	0 (0.00)	1 (2.63)	

Values are presented as mean (standard deviation) or No. (%). *P* values were calculated from t test or Wilcoxon test for continuous variable, and from Chi-square test or Fisher exact test for categorical variables. hs-cTnI, high-sensitivity cardiac troponin I; HR, heart rate; RR, respiration rate; MAP, mean arterial pressure; pH, potential of Hydrogen; pO2, partial pressure of oxygen in blood; and pCO2, partial pressure of carbon dioxide in blood.

Table S3. hs-cTnI levels and characteristics of 244 patients with COVID-19

	hs-cTnI normal	hs-cTnI elevated	
	(N=199, hs-cTnI≤20ng/L)	(N=45, hs-cTnI＞20ng/L)	*P* value
Lymphocyte count ($\times10^9$/L)[a]	1.00 (0.72 - 1.50)	0.74 (0.59 - 1.05)	< 0.001
Neutrophil count ($\times10^9$/L)[a]	3.34 (2.30 - 5.57)	5.03 (2.91 - 6.25)	0.01
CD4 (/μL)[a]	418.00 (236.50 - 610.00)	272.00 (181.00 – 389.50)	< 0.001
CD8 (/μL)[a]	229.00 (135.00 - 359.00)	135.00 (92.50 – 218.00)	0.001
Haemoglobin (g/L)[b]	125.14 ± 15.46	127.20 ± 14.77	0.41
Platelet count ($\times10^9$/L)[a]	238.00 (174.00 - 291.50)	200.00 (151.00 - 241.00)	0.01
<100, No.(%)	7 (3.52)	4 (8.89)	0.12
≥100, No.(%)	192 (96.48)	41 (91.11)	
C-Reactive Protein (mg/L)[a]	24.00 (5.00 - 71.10)	72.40 (42.00 - 140.00)	< 0.001
Procalcitonin (ng/mL), No.(%)	0.05 (0.03 - 0.12)	0.15 (0.07 - 0.33)	< 0.001
< 0.1, No.(%)	144 (72.36)	16 (35.56)	< 0.001
≥ 0.1, No.(%)	55 (27.64)	29 (64.44)	
CK-MB (ng/ml)[a]	0.90 (0.64 - 1.35)	1.98 (1.29 - 2.80)	< 0.001
Myoglobin (μg/L)[a]	37.30 (25.85 - 57.50)	85.51 (45.40 - 177.00)	< 0.001
NT-proBNP (pg/ml)[a]	121.55 (33.50 - 245.45)	614.65 (384.47 - 1316.50)	< 0.001
eGFR (ml/min/1.73 m^2)[a]	96.99 (90.00 - 95.97)	88.35 (77.27 - 83.12)	< 0.001
Alanine aminotransferase (U/L)[a]	28.00 (17.00 - 47.00)	26.00 (18.00 - 36.00)	0.46
Aspartate aminotransferase (U/L)[a]	30.00 (19.50 - 42.00)	39.00 (29.00 - 54.00)	0.002
≤40 U/L, No.(%)	145 (72.86)	24 (53.33)	0.02
> 40 U/L, No.(%)	54 (27.14)	21 (46.673)	
Alkaline phosphatase(U/L)[a]	64.00 (52.00 - 78.00)	64.00 (51.00 - 100.00)	0.49
Gamma glutamyl transpeptidase(U/L)[a]	31.00 (19.50 - 57.50)	27.00 (21.00 - 49.00)	0.70
Total bilirubin(μmol/L)[a]	10.70 (8.05 - 14.70)	12.90 (10.50 - 16.10)	0.004
Conjugated bilirubin(μmol/L)[a]	3.90 (2.80 - 5.40)	5.40 (4.30 - 6.70)	< 0.001
Serum creatinine(μmol/L)[a]	63.00 (52.00 - 72.00)	65.00 (48.00 - 77.00)	0.51
Random blood glucose (mmol/L)[a]	5.60 (4.80 - 7.05)	5.70 (5.10 - 8.00)	0.24
Potassium (mmol/L)[a]	4.00 (3.69 - 4.40)	3.69 (3.48 - 4.11)	0.009
Sodium (mmol/L)[a]	141.00 (138.00 - 144.00)	141.00 (136.00 - 144.00)	0.31
Triglyceride (mmol/L)[a]	1.23 (0.95 - 1.67)	1.38 (1.00 - 1.97)	0.18
Total cholesterol (mmol/L)[a]	3.81 (3.27 - 4.50)	3.80 (3.39 - 4.27)	0.96
Creatine kinase (U/L)[a]	58.00 (37.00 - 92.00)	105.00 (58.00 - 185.00)	< 0.001
Lactate dehydrogenase (U/L)[a]	268.00 (202.00 - 358.50)	408.00 (325.00 - 545.00)	< 0.001
≤ 250, No. (%)	89 (44.72)	2 (4.44)	< 0.001
> 250, No. (%)	110 (55.28)	43 (95.56)	

Values are presented as [a]median (interquartile range) , No. (%) or [b]mean (standard deviation). *P* values were calculated from t test or Wilcoxon test for continuous variable, and from Chi-square test or Fisher exact test for categorical variables. hs-cTnI, high-sensitivity cardiac troponin I; NT-proBNP, N-terminal pro-B-type natriuretic peptide; and CK-MB, creatinine kinase-myocardial band.

Figure S1.

ROC curves for the classification of critical medical condition.

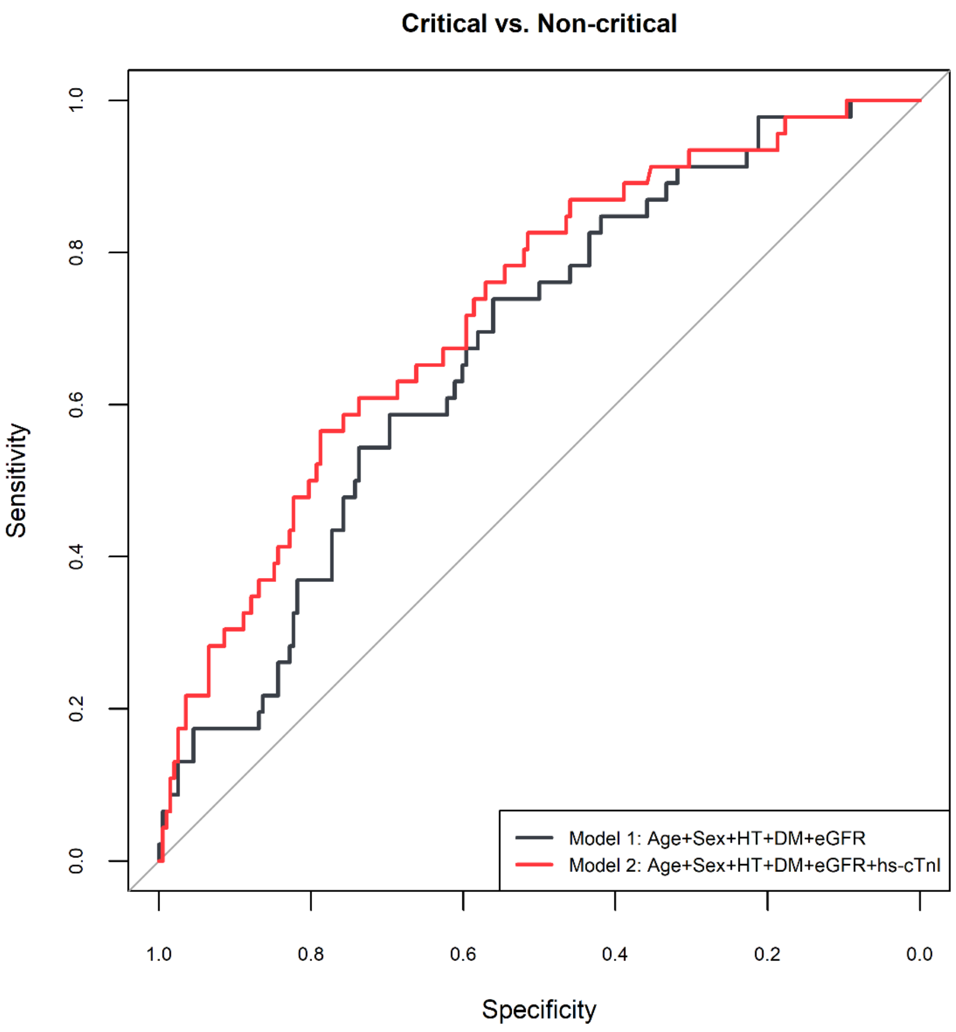

Model 1 is the basic model, included age, sex, hypertension, diabetes, and eGFR, with an AUC1 =0.673 (95% CI: 0.592-0.754); Model 2: Model 1 + hs-cTnI (log), AUC2 = 0.725 (95% CI: 0.645-0.804); *P*=0.06 for the difference between AUC1 and AUC2.

ROC, receiver operating characteristic; AUC, area under the curve; eGFR, estimated glomerular filtration rate; and hs-cTnI, high-sensitivity cardiac troponin I.

Figure S2.

ROC curves for the prediction of in-hospital fatality according to the admission high-sensitivity cardiac troponin I levels.

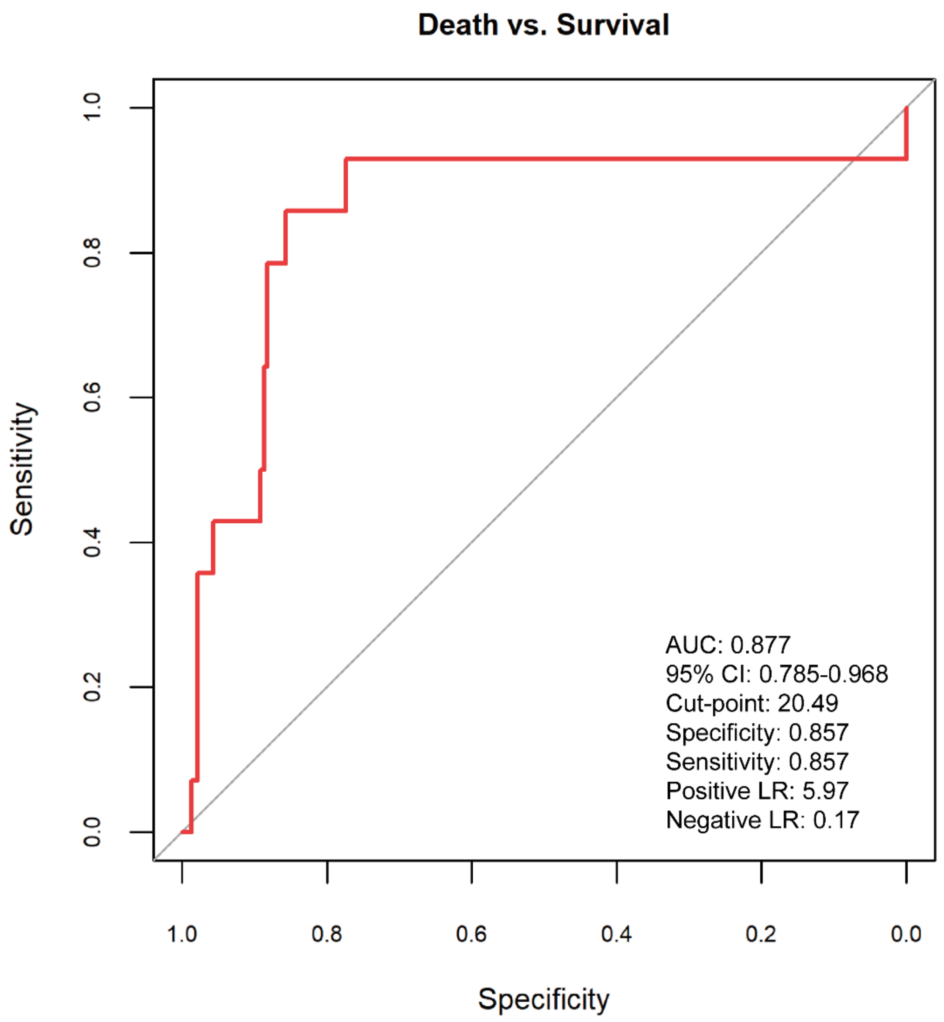

ROC, receiver operating characteristic; AUC, area under the ROC curve; CI, confidence interval; and LR, likelihood ratio.

Supplementary Method

Clinical severity Classifications of COVID-19 patients in China

1.Mild Cases

The clinical symptoms are mild, and no pneumonia manifestation can be found in imaging.

2.Moderate Cases

Patients have symptoms like fever and respiratory tract symptoms, etc. and pneumonia manifestation can be seen in imaging.

3.Severe Cases

Meeting any of the following:

- Respiratory distress, RR >30 breaths/min;
- The oxygen saturation is less than 93% at a rest state;
- Arterial partial pressure of oxygen (PaO_2) / oxygen concentration (FiO_2) <300 mmHg (1 mmHg=0.133 kPa). For high altitude areas (above 1 kilometer), PaO_2/PiO_2 values should be adjusted based on the equation of $PaO_2 \times$ [Atmospheric Pressure (mmHg)/760].
- Patients with >50% lesions progression within 24 to 48 hours in pulmonary imaging should be treated as severe cases.

4.Critical Cases

Meeting any of the following :

- Respiratory failure occurs, and mechanical ventilation is required;
- Shock occurs;
- Complicated with other organ failures that require monitoring and treatment in ICU.

*Guidelines on the Novel Coronavirus-Infected Pneumonia Diagnosis and Treatment (Provisional 6th Edition) issued by China's National Health Commission.

New coronavirus pneumonia prevention and control program (6th ed) (in Chinese). 2020. http://www.nhc.gov.cn/yzygj/s7653p/202002/8334a8326dd94d329df351d7da8aefc2/files/b218cfeb1bc54639af227f922bf6b817.pdf.
